# Mapping histoplasmosis in South East Asia – implications for diagnosis in AIDS

**DOI:** 10.1080/22221751.2019.1644539

**Published:** 2019-07-31

**Authors:** Jacob Baker, Findra Setianingrum, Retno Wahyuningsih, David W. Denning

**Affiliations:** a The University of Manchester and the Manchester Academic Health Service Centre, Manchester, UK; b Faculty of Medicine, Universitas Indonesia, Jakarta, Indonesia; c Faculty of Medicine, Universitas Kristen Indonesia, Jakarta, Indonesia; d The National Aspergillosis Centre, Education and Research Centre, Wythenshawe Hospital, Manchester University NHS Foundation Trust, Manchester, UK

**Keywords:** Histoplasma, AIDS, adrenal, disseminated, skin testing

## Abstract

Histoplasmosis caused by the fungus *Histoplasma capsulatum* is often lethal in patients with AIDS. Urine antigen testing is highly sensitive and much quicker for diagnosis than culture. Histoplasmosis has a patchy and incompletely appreciated distribution around the world especially in South East Asia. We conducted a systematic literature review of cases of all disease forms of histoplasmosis in SE Asia, not including the Indian sub-continent. We also reviewed all histoplasmin skin test mapping studies to determine localities of exposure. We found a total of 407 cases contracted or likely to have been contracted in SE Asia. Numbers of cases by country varied: Thailand (233), Malaysia (76), Indonesia (48) and Singapore (21), with few or no cases reported in other countries. Most cases (255 (63%)) were disseminated histoplasmosis and 177 (43%) cases were HIV associated. Areas of high histoplasmin skin test sensitivity prevalence were found in Myanmar, the Philippines, Indonesia, Thailand and Vietnam - 86.4%, 26.0%, 63.6%, 36.0% and 33.7%, respectively. We have drawn maps of these data. Further study is required to ascertain the extent of histoplasmosis within SE Asia. Diagnostic capability for patients with HIV infection is urgently required in SE Asia, to reduce mortality and mis-diagnosis as tuberculosis.

## Introduction

Histoplasmosis was first described in 1906 by Dr Samuel Darling in Panama during the United States’ construction of the canal [1]. In the following decades *Histoplasma capsulatum* var *capsulatum* (Hcc) was conclusively proved to be the aetiological agent of histoplasmosis [2] and found in soil, and then bird and bat excrement [3–5]. Hcc is a thermally dimorphic fungus that grows as a mycelium in the environment whilst developing into a small intracellular yeast in the body [6].

Disseminated histoplasmosis is an AIDS-defining infection (and often referred to as progressive disseminated histoplasmosis), and co-infection with tuberculosis in AIDS patients presents further clinical challenges [7]. HIV infection with very low CD4 counts in endemic areas leads to disseminated histoplasmosis [8]. Reports of histoplasmosis in AIDS emerged from areas in parts of the world where histoplasmosis had previously seldom or never been reported, such as Trinidad [9], Thailand [10] and the Democratic Republic of Congo [11]. The identification of cases in supposedly non-endemic and entirely novel regions as a consequence of the HIV epidemic, assisted in the realization that Hcc was far more widespread than initially thought [12]. Other immunocompromised states also pose a risk including solid organ transplantation [13] and the recently described Adult Immunodeficiency Syndrome [14]. Those without immunocompromise may develop acute pulmonary histoplasmosis or subacute disseminated histoplasmosis, typically with lymph node, adrenal gland or gastro-intestinal involvement.

Most epidemiological data concerning histoplasmosis comes from the United States which has a wider geographical distribution than previously appreciated [15]. Recently collated older studies show the patchy distribution of Hcc in various areas around the world [3,16] with case series in Brazil [17], South Africa [18], India [19] and sporadic cases in Central and South America, northern sub-Saharan Africa, Oceania, and Europe [20]. Four recent reviews have documented the disease distribution and burden in Latin America, China, Africa and the Indian subcontinent [19,21–23]. In China 300 cases were described from 1990 to 2011 with 75% of cases found along the course of the Yangtze river [21]; disseminated histoplasmosis in AIDS formed a clear majority of cases as opposed to pulmonary infection. The recent review of 470 cases of histoplasmosis in Africa over the period 1952–2017 [22] included *Histoplasmosis capsulatum* subtype *duboisii*, “African histoplasmosis” [24]. *H. duboisii* formed the majority of cases in West Africa, but in Southern Africa Hcc predominated causing 119 of 150 cases. HIV infection existed in 38% of cases, and in HIV positive patients, the disease was more likely to be disseminated [22]. In India, 388 cases were published from 1995 to 2017, of whom most were diagnosed from 2004 onwards, and overall 29% were HIV-associated [19]. Throughout Latin America, the number of deaths from histoplasmosis in AIDS exceeds that from TB – an estimate of 6710–15,657 cases in 2012 and 671–9394 deaths [23].

Historically Hcc epidemiology has been neglected in South East Asia despite a review in 1970 by H. S. Randhawa that found a total of 30 autochthonous cases, half being culture confirmed, in Malaysia, Indonesia, Singapore, Thailand, South Vietnam, India and Japan [25]. Hcc was found in bat guano in Malaysia and prior exposure was common as measured by skin sensitivity [26]. The one regional exception is Thailand; its Ministry of Public Health reported 1253 disseminated histoplasmosis cases among HIV infected patients from September 1984 to March 2010 [27]. We set out to map the localities with documented cases and exposure to Hcc in SE Asia, not including the Indian Sub-continent.

## Materials and methods

Searches were made of the literature in several databases and search engines: Medline, PubMed, Cochrane library and Google Scholar, without date restriction, and conducted in June 2018. The Medline and PubMed searches were performed using the Health Database Advanced Search software (HDAS) produced by the National Institute for Clinical Evidence (NICE). This search was performed using the key words “histoplasmosis” and “South East Asia” using the HDAS explode function to include the names of each individual South East Asian country and significant geographical regions such as “Borneo”. The following nations were included as part of South East Asia: Cambodia, Brunei, East Timor, Indonesia, Laos, Malaysia, Myanmar, Pacific Islands, Philippines, Singapore, Thailand, Vietnam. The Pacific Islands representing the collection of small island territories in eastern South East Asia. These key words were appropriately linked using the Boolean operators “AND” and “OR”. The search of the two databases produced a total of 123 individual publications (Figure S1). While many of these publications discussed histoplasmosis without presenting new cases, a total of 320 relevant individual cases were found. The search of the Cochrane library produced no relevant results. The search using the Google Scholar engine was performed with the key term “histoplasmosis” and “South East Asia” and the individual country names in a similar fashion with the addition of the key word “cases” in order to further narrow the results. This search produced over 7000 results and only the top 500 hits were reviewed as fewer and fewer pertinent papers appeared after that point. Once results that had already been seen in the PubMed and Medline searches were removed the Google Scholar search had produced a further 53 relevant individual reported cases. Additional Indonesian cases were found by a thorough manual scanning of the bibliographies listed in selected articles in the Indonesian language, as well as annual reports of the Ministry of Health and an additional 10 cases provided by author RW which were presented by poster at International Society of Health and Medicine conference 2015, Melbourne Australia. We omitted the 1253 Thai cases reported by the Ministry of Health [27], through lack of detail.

In terms of inclusion criteria, only cases in which the country of origin was clear were included, the cases country of origin having been explicitly stated in the paper or otherwise made clear in the publication. Cases from outside South East Asia where the location of initial infection was noted to be in South East Asia were included. Cases were included whether they were diagnosed by positive culture or by other means and meeting the Mycoses Study Group (MSG) diagnostic criteria [28].

The results were broken down by country, whether disease was progressive disseminated disease and by the presence or absence of HIV co-infection. The disease form was recorded if explicitly noted in the publication or obvious by the case discussion and description. Some publications, noted as “Mixed” concerning disease form or HIV status in the full record of cases in Appendix 1, reported multiple cases of various disease forms and HIV status. If case by case details were given within the article this data was used to break down the study to report the number of cases with HIV co-infection and disseminated disease (See Table 1).

**Table 1.** Literature review of cases of histoplasmosis in South East Asia from 1932 to 2018.

Country	Number of cases	Number of cases with HIV co-infection	Number of cases of disseminated disease
Cambodia	5	5	4
Brunei	0	0	0
East Timor	0	0	0
Indonesia	48	24	28
Laos	1	0	0
Malaysia	76	8	21
Myanmar	3	2	0
Pacific Islands	1	0	0
Philippines	14	0	13
Singapore	21	9	14
Thailand	233	129	172
Vietnam	5	0	3
Total	407	177	255

The prevalence of histoplasmin skin sensitivity provides an alternative data source for mapping exposure to Hcc. Histoplasmin is an antigenic extract from the fungus that can be inoculated into the skin and examined for immunological response to test for sensitization suggesting prior exposure in a similar fashion to the Mantoux test for tuberculosis.

## Results

**Figure 1.**
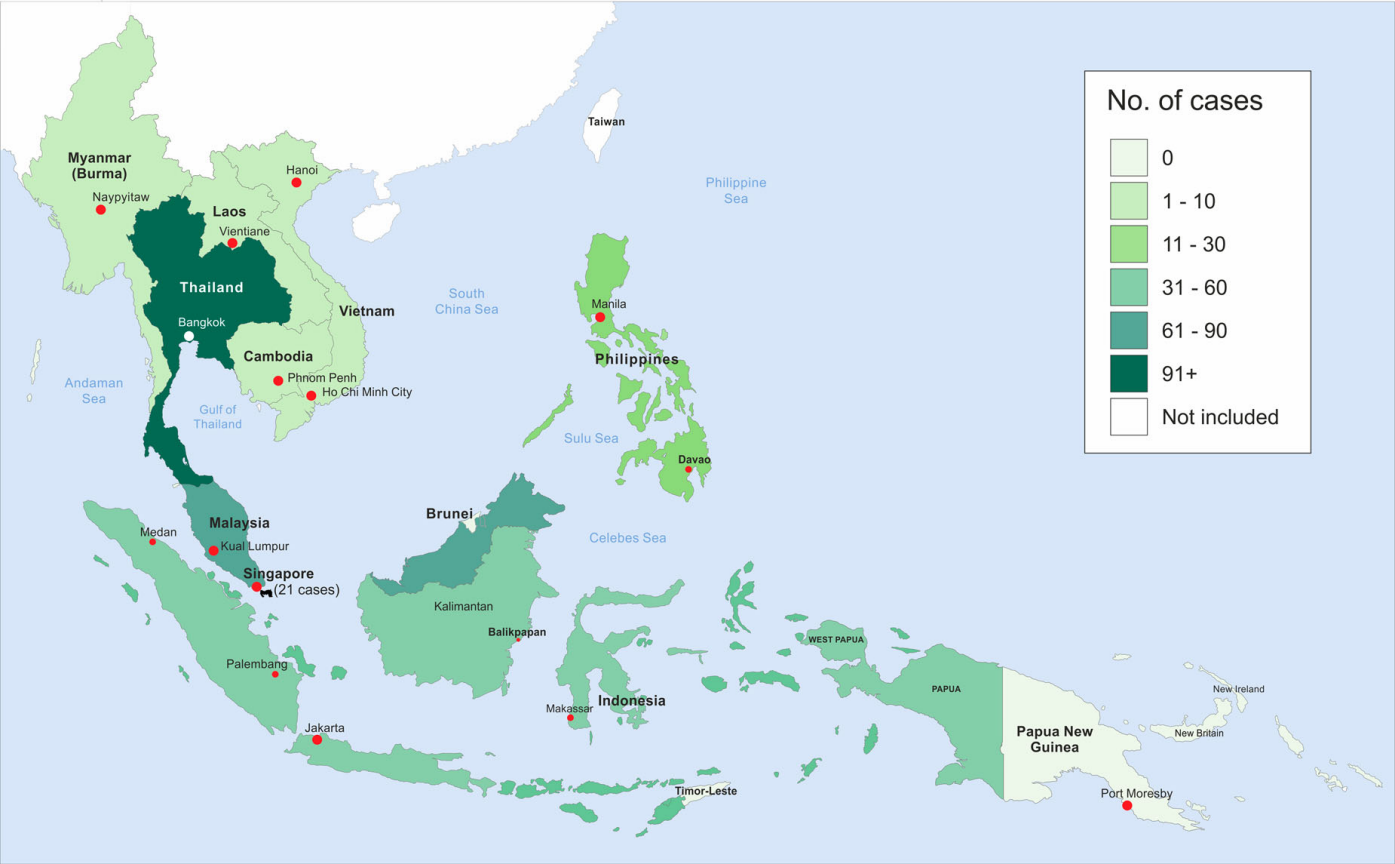
Map of reported cases of histoplasmosis infection by country 1932–2018. This map excludes 1253 disseminated histoplasmosis cases among HIV infected patients from September 1984 to March 2010, partly because of uncertainty about duplication.

Our review of the literature produced a total of 407 cases of histoplasmosis reported in SE Asia or in which infection had occurred or was most likely to have occurred in SE Asia. The only countries included in the search to not produce any cases were Brunei and East Timor. The largest number of cases by far was found in Thailand (233) followed by Malaysia (76), Indonesia (48) and Singapore (21). The remaining countries had a relatively small number of cases reported (Table 1). Disseminated histoplasmosis was the single most common disease form comprising 255 out of the total 407. Most cases (255 (63%)) were disseminated histoplasmosis and 177 (43%) cases were HIV-associated. Our review of cases is presented cartographically below (Figure 1).

An additional four cases were reported as potential cases, but infection was more or equally likely to have been in other areas outside South East Asia (See Appendix 1: “Potential” cases).

Two detailed case reviews from Kuala Lumpur in Malaysia [29] and Bangkok in Thailand [30] examined the relative proportions of different opportunistic infections in AIDS. The Kuala Lumpur review took place throughout 2001 and 2002 while the Siriraj review was throughout 2002. Opportunistic infections in HIV-infected patients are summarized in Table 2. In Kuala Lumpur, 1.5% of patients had histoplasmosis while 1% of patients in Bangkok had histoplasmosis. In the Kuala Lumpur study only AIDS patients admitted to the hospital were included, whereas in Bangkok 11 HIV positive patients, were included (average CD4 count of 74.7 ± 134.21 cells/mm^3^).

**Table 2.** A summary of the results of retrospective reviews of cohorts of HIV positive patients in Kuala Lumper Hospital Malaysia [29] and Siriraj Hospital Thailand [30].

	Kuala Lumpur	Bangkok
Years reviews	2001–2002	2002
HIV patient cohort size	205	286
Patients on anti-retroviral therapy	86%	7.7%
Patients with histoplasmosis	1.5%	1%
Patients with tuberculosis	45%	29%
Patients with *Pneumocystis*	12%	19%
Patients with cryptococcosis	3%	16%
Patients with toxoplasmosis	9%	6%

The non-HIV associated cases were reviewed for any further host risk factors explicitly stated in the case reports, the results shown in Table 3. The most commonly reported risk factors were diabetes mellitus (DM) (6), age under 13 (5) or renal/liver transplant recipient (4) however this is a likely gross underreporting of non-HIV risk factors as many cases, particularly in larger case series, fail to adequately report individual patient backgrounds. A full list of cases found is available in Appendix 1.

**Table 3.** Cases with identified host risk factors excluding HIV infection.

Host risk factor	No. of cases
Diabetes mellitus	6
Renal/liver transplant	4
Young age (<13 years)	5
Corticosteroid therapy	2
Connective tissue disease (e.g. SLE)	2
Concurrent amoebic colitis	1
Other immunosuppressive disease	2
Total	22

**Figure 2.**
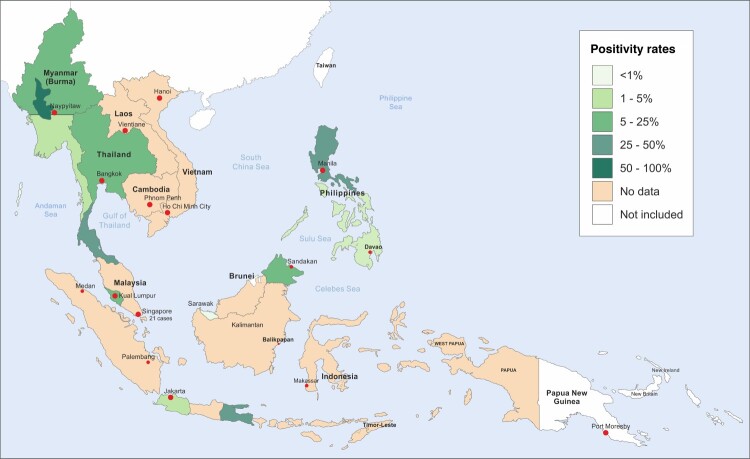
Map showing the highest reported histoplasmin sensitivities in South East Asia.

Histoplasmin sensitivities for South East Asia are summarized below in Table 4 and displayed cartographically in Figure 2. Data from Myanmar was published in 1952 after testing 3558 people in different parts of what was then Burma. However, the testing was carried out in prison populations rather than the public. Upper and Lower Myanmar had positivity rates of 8.4% and 14.5%, respectively. Areas of hyperendemicity were suggested by the high positivity rates in Rangoon and Maguee, 27.1% and 86.4%, respectively [31]. The data from the Philippines come from a 1964 study of 2577 United States Naval recruits in Manila, where 6.4% of recruits were deemed to have a positive reaction of over 6 mm [32], and a second public health study in 2000 reported 26% positivity in Luzon Island amongst 143 electrical company workers [33]. Our data from Thailand comes from two sources, firstly a 1967 study of 497 healthcare students in Siriraj hospital in Bangkok which showed a positivity rate of 5.6% [34]. The second was a large 1968 multicentre study involving 4,211 Thai prisoners, naval recruits and other groups. Positivity ranged from 4.8 to 34.4% with a total average of 13.8% [35]. The researchers produced ranges and averages for Thailand divided into North, Central and Eastern regions as displayed in our cartography. Concerning Indonesia, a 1956 paper reported 2542 residents of Jakarta, a mix of students, nurses and adult patients, showed a positivity of 12.5% and 2.7% in children positivity being an induration of 6 mm or more [36]. Edwards et al. in 1956 produced data for both Surabaya and Kedisan in Indonesia and the then-named Saigon in Vietnam in a large study covering parts of Africa and South Asia totalling 10,000 test subjects, primarily children. The cities produced positivity rates of 32.0, 63.6 and 33.7%, respectively [37]. A 1997 study in Medan in Indonesia of 169 medical students found a positivity rate of 13.6% [38]. The third division of Sarawak was studied in 1963. Of the 183 school children tested 0.5% were positive [39]. In a Malaysia, a 1964 study of 227 patients from 5 to 60 years of age revealed the presence of positive histoplasmin skin tests in 10.5% [26]. An additional study in 1971 in the east Malaysian state of Sabah showed positivity rates of 11.8% in 3824 people tested [40]. All studies, unless stated otherwise, considered an induration of 5 mm or more as positive.

**Table 4.** Histoplasmin positivity rates in studies in South East Asia.

No	Area	Percent positive (%)	Dilution used	Population studied	Reference
1	Myanmar (Burma)- Upper	8.4	1:100	3558 prisoners/ prison staff	[31]
2	Myanmar (Burma)- Lower	14.5			[31]
3	Myanmar (Burma)- Rangoon	27.1			[31]
4	Myanmar(Burma)- Maguee	86.4			[31]
5	Philippines- Manila	6.4	1:100	2577 navy recruits	[32]
6	Philippines-Luzon Island	26.0	*	143 electric company employees	[33]
7	Thailand-Bangkok	5.6	N/A	497 medical/nursing students	[34]
8	Thailand-Northern region	14.0	1:100	4211 prisoners	[35]
9	Thailand-Central region	9.0			[35]
10	Thailand-Southern region	36.0			[35]
11	Indonesia-Jakarta (adults/children)	12.5/2.7	N/A	2542 students/nurses/hospital patients	[36]
12	Indonesia-Surabaya	32.0	N/A	281 school children/villagers	[37]
13	Indonesia-Kedisan (Bali)	63.6	1:100	340 school children/villagers	[37]
14	Indonesia-Medan	13.6	1:200	1265 medical Student	[38]
15	Malaysia-Sarawak	0.5	1:100	181 school children/hospital patients	[39]
16	Vietnam-Saigon	33.7	1:100	303 school children/villagers	[37]
17	Malaysia-Kuala Lumpur	10.5	N/A	224 adult residents	[26]
18	Malaysia-Sabah	11.8	N/A	3824 adult residents	[40]

*Histolyn-CYL.

N/A – Information not available.

## Discussion

The principal limitation of comparing the results is that they are affected by the varying standard of health services within each individual country and their efforts in publishing cases. Healthcare resources of each country vary substantially including their appreciation of histoplasmosis as a significant health concern. Several factors including economic and standards of clinician education and governmental organization impact of the ability to diagnose histoplasmosis. Additionally, due to the extended time in which the cases were reporting and the variation in population sizes of each country it would not be sufficiently valid to produce per capita comparisons. We take the view that these results should not be used to make direct comparisons between nations but rather as evidence to support the endemicity of histoplasmosis within each individually.

One of the factors that appears to have created the disparity between Thailand and Malaysia and the other South East Asian countries in terms of the number of cases is varying attention of public health services to histoplasmosis in each country. In Thailand, in particular, there have been several large retrospective cohort studies of HIV positive patients with active case finding of opportunistic infections such as histoplasmosis [41,42]. Similar large retrospective reviews have been performed in Malaysia [43]. These studies have largely been carried out in the last two decades. Because of these studies, which are lacking in other South East Asian countries, higher numbers of cases reported in Thailand and Malaysia may in part be due to increased levels of clinical awareness rather than an actual increased prevalence of the disease in those places. In Thailand and Malaysia, the HIV-associated burden of histoplasmosis has been calculated at 184 and 175 for Thailand [44] and Malaysia [45] respectively, indicative of major under-reporting of cases. These studies demonstrated the potentially high mortality of histoplasmosis in AIDS; for example Wongprommek reported a survival rate at 6- and 12-month of 88% and 75%, respectively, out of 57 cases, not all HIV positive [42].

We have used these studies to draw more direct comparisons between these two countries, or at least particular areas of those countries. While histoplasmosis is considered a significant opportunistic infection in AIDS it was found relatively rarely compared to other important infections (See Table 2). Published in 2002 and 2004, these papers [29,30] do not describe diagnostic procedures in detail, but are prior to the first description of *Histoplasma* PCR and availability of antigen outside the USA. This could reflect lack of awareness of clinicians about histoplasmosis but also lack of more sensitive diagnostic tools such as antigen detection or direct PCR detection, as applied in Latin America [46–48]. A review of laboratories in the South Asia region found that 78.9% of laboratories lack histoplasmosis antigen testing capabilities [49].

Our review noted 255 cases of disseminated disease, either explicitly reported or as made clear by case discussion, out of the total of 407. However, this should not be taken to represent the actual proportions of this disease manifestation in South East Asia. One reason for this is that many of the cases found in this study come from reviews of cohorts of HIV positive patients [29,30] in whom histoplasmosis is more likely to present as disseminated disease [21]. These reviews, with their selection of patients to fit their research purposes, skews the number of cases towards more disseminated disease. Additionally, as primary pulmonary histoplasmosis is usually asymptomatic or mild it will go largely unreported. The chronic pulmonary form of the disease is easily misdiagnosed as tuberculosis [50,51] leading to lack of recognition and under reporting. Therefore, the high percentage of disseminated disease in our literature should not be taken as representative of the disease pattern in South East Asia.

In addition to the epidemiological study of cases of histoplasmosis, the prevalence of histoplasmin skin sensitivity can be examined and can help determine the endemicity of histoplasmosis in South-East Asia [50]. Histoplasmin sensitization is a useful proxy for prior exposure and has been used extensively in the Americas, but less so in other parts of the world [12,16,22,23]. The histoplasmin test can show very high levels of sensitivity in areas known to be endemic such as parts of the United States, Guatemala, Mexico with population reactivity rates up to 90% [16]. Positivity rates are epidemiologically useful in the study of histoplasmosis. The global rate of histoplasmin positivity rate is 5–14%, while it may reach 80–90% in the hyperendemic areas [51]. The rate of histoplasmin test reactivity was 13.6% among the healthy population in Indonesia and there was a significant correlation between reactivity and animal, presence of bat, cave adventure and history of visiting forest area [38]. A large epidemiological study of histoplasmin in Myanmar showed the positivity rate of 27.1% among prison population and their families [31]. Myanmar, the Philippines, Indonesia, Thailand and Vietnam showed areas with high rates of histoplasmin sensitivity with areas showing 86.4%, 26.0%, 63.6%, 36.0% and 33.7%, respectively. These reactivity rates are above the global rate of histoplasmin sensitivity which support the endemicity of histoplasmosis in South-East Asia. One uncertainty is whether there is any cross-reactivity with *Talaromyces* (*Penicillium*) *marneffei*, which we do not believe has been studied. We believe further up to date testing is required in the region and it is critical it be made available to local health authorities. With this information safety precautions amongst at risk individuals such as the use of protective equipment and the clearance of bird and bat droppings as is being developed in the USA [52,53].

## Conclusions

Histoplasmosis is endemic in multiple regions within South East Asia and further study is required to ascertain the extent of this. Exposure data are absent for many areas. Hyperendemic regions of histoplasmosis include the central area of Myanmar, southern Thailand, northern Philippines and certain localized areas of Indonesia. Thailand regularly diagnoses disseminated histoplasmosis in AIDS, unlike most of the other countries in the region. Diagnostic capability for patients with HIV infection is urgently required in South East Asia, to reduce mortality and mis-diagnosis as tuberculosis.

## Supplementary Material

Supplemental Material

## References

[1] Darling. A protozoon general infection producing pseudotubercles in the lungs. J Am Med Assoc. 1906;46:1283–1285.

[2] DeMonbreun. The cultivation and cultural characteristics of Darling's histoplasma capsulatum. The American Society of Tropical Medicine and Hygiene. 1934.

[3] Cano MV, Hajjeh RA. The epidemiology of histoplasmosis: a review. Semin Respir Infect. 2001;16(2):109–118. doi: 10.1053/srin.2001.24241 11521243

[4] Taylor RL, Shacklette MH, Kelley HB. Isolation of histoplasma capsulatum and Microsporum gypseum from soil and bat guano in Panama and the canal Zone. Am J Trop Med Hyg. 1962;11:790–795. doi: 10.4269/ajtmh.1962.11.790 13980368

[5] Emmons CW. Isolation of histoplasma capsulatum from soil. Public Health Rep. 1949;64(28):892–896. doi: 10.2307/4587021 18134389

[6] Maresca B, Kobayashi GS. Dimorphism in histoplasma capsulatum: a model for the study of cell differentiation in pathogenic fungi. Microbiol Rev. 1989;53(2):186–209. 2666842 10.1128/mr.53.2.186-209.1989 PMC372727

[7] Couppie P, Aznar C, Carme B, et al. American histoplasmosis in developing countries with a special focus on patients with HIV: diagnosis, treatment, and prognosis. Curr Opin Infect Dis. 2006;19(5):443–449. doi: 10.1097/01.qco.0000244049.15888.b9 16940867

[8] Wheat LJ, Slama TG, Zeckel ML. Histoplasmosis in the acquired immune deficiency syndrome. Am J Med. 1985;78(2):203–210. doi: 10.1016/0002-9343(85)90427-9 3871588

[9] Bartholomew C, Raju C, Patrick A, et al. AIDS on trinidad. Lancet. 1984;1(8368):103. doi: 10.1016/S0140-6736(84)90029-1 6140399

[10] Vithayasai P, Vithayasai V. Clinical manifestations of 174 AIDS cases in maharaj nakorn chiang mai hospital. J Dermatol. 1993;20(7):389–393. doi: 10.1111/j.1346-8138.1993.tb01305.x 8408918

[11] Geffray L, Veyssier P, Cevallos R, et al. [African histoplasmosis: clinical and therapeutic aspects, relation to AIDS. Apropos of 4 cases, including a case with HIV-1-HTLV-1 co-infection]. Ann Med Interne (Paris). 1994;145(6):424–428. 7864504

[12] Antinori S. Histoplasma capsulatum: more widespread than previously thought. Am J Trop Med Hyg. 2014;90(6):982–983. doi: 10.4269/ajtmh.14-0175 24778192 PMC4047757

[13] Nieto-Rios JF, Serna-Higuita LM, Guzman-Luna CE, et al. Histoplasmosis in renal transplant patients in an endemic area at a reference hospital in Medellin, Colombia. Transplant Proc. 2014;46(9):3004–3009. doi: 10.1016/j.transproceed.2014.06.060 25420811

[14] Browne SK, Burbelo PD, Chetchotisakd P, et al. Adult-onset immunodeficiency in Thailand and Taiwan. N Engl J Med. 2012;367(8):725–734. doi: 10.1056/NEJMoa1111160 22913682 PMC4190026

[15] Benedict K, Thompson GR 3rd, Deresinski S, et al. Mycotic infections acquired outside areas of known endemicity, United States. Emerg Infect Dis. 2015;21(11):1935–1941. doi: 10.3201/eid2111.141950 26485441 PMC4622235

[16] Bahr NC, Antinori S, Wheat LJ, et al. Histoplasmosis infections worldwide: thinking outside of the Ohio river valley. Curr Trop Med Rep. 2015;2(2):70–80. doi: 10.1007/s40475-015-0044-0 26279969 PMC4535725

[17] Londero AT, Fischman O, Ramos C. A critical review of medical mycology in Brazil 1946-1960. Mycopathol Mycol Appl. 1962;1;18(4):293–316. doi: 10.1007/BF02051452 14059712

[18] Klugman HB, Lurie HI. Systemic histoplasmosis in South Africa. A review of the previous cases and a report of an additional case–the first successfully treated. S Afr Med J. 1963;37:29–31. 14033690

[19] Randhawa HS, Gugnani HC. Occurrence of histoplasmosis in the Indian Sub-continent: An Overview and Update. J Med Res Pract. 2018;7:71–83.

[20] Mochi A, Edwards PQ. Geographical distribution of histoplasmosis and histoplasmin sensitivity. Bull World Health Organ. 1952;5(3):259–291. 14935779 PMC2554039

[21] Pan B, Chen M, Pan W, et al. Histoplasmosis: a new endemic fungal infection in China? review and analysis of cases. Mycoses. 2013;56(3):212–221. doi: 10.1111/myc.12029 23216676

[22] Oladele RO, Ayanlowo OO, Richardson MD, et al. Histoplasmosis in Africa: An emerging or a neglected disease? PLoS Negl Trop Dis. 2018;12(1):e0006046. doi: 10.1371/journal.pntd.0006046 29346384 PMC5773084

[23] Adenis AA, Valdes A, Cropet C, et al. Burden of HIV-associated histoplasmosis compared with tuberculosis in Latin America: a modelling study. Lancet Infect Dis. 2018;18(10):1150–1159. doi: 10.1016/S1473-3099(18)30354-2 30146320 PMC6746313

[24] Gugnani HC, Muotoe-Okafor F. African histoplasmosis: a review. Rev Iberoam Micol. 1997;14(4):155–159. 15538817

[25] Randhawa HS. Occurrence of histoplasmosis in Asia. Mycopathol Mycol Appl. 1970;41(1):75–89. doi: 10.1007/BF02051485 4938836

[26] Ponnampalam JT. Histoplasmosis in Malaya. Br J Dis Chest. 1964;58:49–55. doi: 10.1016/S0007-0971(64)80032-2 14152216

[27] Norkaew T, Ohno H, Sriburee P, et al. Detection of environmental sources of Histoplasma capsulatum in Chiang Mai, Thailand, by nested PCR. Mycopathologia. 2013;176(5–6):395–402. doi: 10.1007/s11046-013-9701-9 24030846

[28] De Pauw B, Walsh TJ, Donnelly JP, et al. Revised definitions of invasive fungal disease from the European organization for research and treatment of cancer/invasive fungal infections cooperative group and the national institute of allergy and infectious diseases mycoses study group (EORTC/MSG) consensus group. Clin Infect Dis. 2008;46(12):1813–1821. doi: 10.1086/588660 18462102 PMC2671227

[29] Nissapatorn V, Lee CK, Rohela M, et al. Spectrum of opportunistic infections among HIV-infected patients in Malaysia. Southeast Asian J Trop Med Public Health. 2004;35(Suppl 2):26–32. 15906630

[30] Anekthananon T, Ratanasuwan W, Techasathit W, et al. HIV infection/acquired immunodeficiency syndrome at Siriraj Hospital, 2002: time for secondary prevention. J Med Assoc Thai. 2004;87(2):173–179. 15061301

[31] Tucker HA, Kvisselgaard N. Histoplasmin and tuberculin sensitivity in Burma: study of tests on 3,558 subjects. Bull World Health Organ. 1952;7(2):189. PMC2554143 13019550

[32] Edwards PQ. Histoplasmin sensitivity of young men in Alaska, Hawaii, the Philippines and Puerto Rico. Bull World Health Organ. 1964;30(4):587. 14178032 PMC2554832

[33] Bulmer AC, Bulmer GS. Incidence of histoplasmin hypersensitivity in the Philippines. Mycopathologia. 2001;149(2):69–71. doi: 10.1023/A:1007277602576 11265164

[34] Prijyanonda B, Bovornkitti S, Oonsombati P, et al. Histoplasmin sensitivity in medical students and student-nurses at Siriraj Hospital. J Med Assoc Thail. 1967;50(1):67–77.

[35] Taylor RL, Duangmani C, Charoenvit Y. The geographic distribution of histoplasmin sensitivity in Thailand. Am J Trop Med Hyg. 1968;17(4):579–583. doi: 10.4269/ajtmh.1968.17.579 5672788

[36] Joe LK, Eng NI, Edwards PQ, et al. Histoplasmin sensitivity in Indonesia1. Am J Trop Med Hyg. 1956;5(1):110–118. doi: 10.4269/ajtmh.1956.5.110 13292657

[37] Edwards PQ, Geser AG, Kjølbye EH, et al. Histoplasmin testing in Africa and southern Asia. Am J Trop Med Hyg. 1956;5(2):224–234. doi: 10.4269/ajtmh.1956.5.224 13302619

[38] Mardianto AT. Prevalensi histoplasmosis pada mahasiswa kedokteran Universitas Islam Sumatera Utara dan hubungan hewan peliharaan dengan tes histoplasmin. Berkala Ilmu Kedokteran. 1997;29:139–144.

[39] Schuman ND, Mackey DM, Safrit HF. Histoplasmin sensitivity investigation in the third division of Sarawak, Borneo. Am Rev Respir Dis. 1963;88(2):261–263. 14045234 10.1164/arrd.1963.88.2.261

[40] Roy RN. Sensitivity to tuberculin, PPD-B and histoplasmin in the population of Sabah in East Malaysia. Med J Aust. 1971;1(6):317–321. 5546216 10.5694/j.1326-5377.1971.tb50271.x

[41] Rangwala F, Putcharoen O, Bowonwatanuwong C, et al. Histoplasmosis and penicilliosis among HIV-infected Thai patients: a retrospective review. Southeast Asian J Trop Med Public Health. 2012;43(2):436–441. 23082594

[42] Wongprommek P, Chayakulkeeree M. Clinical characteristics of histoplasmosis in siriraj hospital. J Med Association of Thailand. 2016;99(3):257–261. 27276735

[43] Ng KH, Siar CH. Review of oral histoplasmosis in Malaysians. Oral Surg Oral Med Oral Pathol Oral Radiol Endod. 1996;81(3):303–307. doi: 10.1016/S1079-2104(96)80330-1 8653464

[44] Chayakulkeeree M, Denning DW. Serious fungal infections in Thailand. Eur J Clin Microbiol Infect Dis. 2017;36(6):931–935. doi: 10.1007/s10096-017-2927-6 28161742

[45] Velayuthan RD, Samudi C, Lakhbeer Singh HK, et al. Estimation of the burden of Serious Human Fungal infections in Malaysia. J Fungi (Basel). 2018;4(1): E38. DOI:10.3390/jof4010038. PMC5872341 29562712

[46] Cáceres DH, Samayoa BE, Medina NG, et al. Multicenter validation of commercial antigenuria reagents to diagnose progressive disseminated histoplasmosis in people living with HIV/AIDS in two Latin American countries. J Clin Microbiol. 2018;56(6):e01959–17. DOI:10.1128/JCM.01959-17. 29563205 PMC5971531

[47] Medina N, Samayoa B, Lau-Bonilla D, et al. Burden of serious fungal infections in Guatemala. Eur J Clin Microbiol Infect Dis. 2017;36(6):965–969. doi: 10.1007/s10096-017-2920-0 28243758

[48] Nacher M, Blanchet D, Bongomin F, et al. Histoplasma capsulatum antigen detection tests as an essential diagnostic tool for patients with advanced HIV disease in low and middle income countries: A systematic review of diagnostic accuracy studies. PLoS Negl Trop Dis. 2018;12(10):e0006802. doi: 10.1371/journal.pntd.0006802 30339674 PMC6209380

[49] Chindamporn A, Chakrabarti A, Li R, et al. Survey of laboratory practices for diagnosis of fungal infection in seven Asian countries: An Asia fungal working group (AFWG) initiative. Med Mycol. 2017;56(4):416–425. doi: 10.1093/mmy/myx066 29036605

[50] Kennedy CC, Limper AH. Redefining the clinical spectrum of chronic pulmonary histoplasmosis: a retrospective case series of 46 patients. Medicine (Baltimore). 2007;86(4):252–258. doi: 10.1097/MD.0b013e318144b1d9 17632267

[51] Unis G, et al. Pulmonary histoplasmosis mimicking tuberculosis. J Bras Pneumol. 2005;31(4):318–324. doi: 10.1590/S1806-37132005000400009

[52] CDC. US Centers for Disease Control and Prevention. Histoplasmosis: Protecting Workers at Risk. Department of Health and Human Services, National Institute of Occupational Safety and Health (NIOSH) Publication No. 2005-109. Atlanta, GA: CDC; 2004.

[53] Diaz JH. Environmental and wilderness-related risk factors for histoplasmosis: more than bats in caves. Wilderness Environ Med. 2018;29(4):531–540. doi: 10.1016/j.wem.2018.06.008 30266238

