# Supplementary material for: Mapping histoplasmosis in South East Asia – implications for diagnosis in AIDS
Source: Emerg Microbes Infect. 2019 Jul 31;8(1):1139–45. doi: 10.1080/22221751.2019.1644539 (PMC6711083; doi:10.1080/22221751.2019.1644539)
Supplement: Supplemental Material [file TEMI_A_1644539_SM4495.zip › Baker_et_al_supplementary_material_.docx]

**Baker et al – supplementary material**

Published cases of histoplasmosis in South East Asia 1932-2018

| Country of Infection | Disease Form | HIV status | Reference |
| --- | --- | --- | --- |
|  |  |  |  |
| Cambodia | Disseminated | Positive | 1 |
| Cambodia | Pulmonary | Positive | 2 |
| Cambodia | Disseminated- 2 cases | Positive | 3 |
| Cambodia | Disseminated | Positive | 4 |
| Indonesia | Mixed- 8 cases | Negative | 5 |
| Indonesia | Disseminated- 2 cases | Negative | 6 |
| Indonesia | Disseminated | Positive | 7 |
| Indonesia | Disseminated- 7 cases | Positive | 8 |
| Indonesia | Disseminated | Negative | 9 |
| Indonesia | Disseminated | Negative | 10 |
| Indonesia | Disseminated | Positive | 11 |
| Indonesia | Laryngeal | Positive | 12 |
| Indonesia | Disseminated | Positive | 13 |
| Indonesia | Disseminated | Negative | 14 |
| Indonesia | Disseminated | Negative | 15 |
| Indonesia | Mixed- 5 cases | Negative | 16 |
| Indonesia | Oral | Negative | 17 |
| Indonesia | Oral | Negative | 18 |
| Indonesia | Disseminated | Positive | 19 |
| Indonesia | Disseminated | Negative | 20 |
| Indonesia | Oral | Negative | 21 |
| Indonesia | Disseminated | Negative | 22 |
| Indonesia | Disseminated- 10 cases | Positive | 23 |
| Potentially Indonesia | Laryngeal | Negative | 24 |
| Indonesia | Cutaneous | Positive | 25 |
| Indonesia | Disseminated | Positive | 26 |
| Laos | Joint infection | Negative | 27 |
| Malaysia | Mixed- 4 cases | Negative | 5 |
| Malaysia | Disseminated | Negative | 28 |
| Malaysia | Disseminated | Negative | 29 |
| Malaysia | Colitis | Negative | 30 |
| Malaysia | Pulmonary- 3 cases | Negative | 31 |
| Malaysia | Disseminated | Negative | 32 |
| Malaysia | Disseminated | Positive | 33 |
| Malaysia | Laryngeal | Negative | 34 |
| Malaysia | Disseminated- 3 cases | Positive | 35 |
| Malaysia | Oral | Negative | 36 |
| Malaysia | Adrenal | Negative | 37 |
| Malaysia | Disseminated | Negative | 38 |
| Malaysia | Disseminated | Positive | 39 |
| Malaysia | Cutaneous- 3 cases | Positive | 40 |
| Malaysia | Oral- 37 cases | Negative | 41 |
| Malaysia | Disseminated | Negative | 42 |
| Malaysia | Disseminated | Negative | 43 |
| Malaysia | Adrenal | Negative | 44 |
| Malaysia | Disseminated- 3 cases | Negative | 45 |
| Malaysia | Disseminated | Negative | 46 |
| Malaysia | Disseminated- 2 cases | Negative | 47 |
| Malaysia | Unknown | Unknown | 48 |
| Malaysia | Disseminated | Negative | 49 |
| Malaysia | Adrenal | Negative | 50 |
| Malaysia | Adrenal | Negative | 51 |
| Malaysia | Adrenal | Negative | 52 |
| Potentially Malaysia | Pulmonary | Negative | 53 |
| Malaysia | Mixed- 2 cases | Negative | 54 |
| Myanmar | Colitis | Positive | 55 |
| Myanmar | Pulmonary | Negative | 56 |
| Myanmar | Meningitis | Positive | 57 |
| Pacific Islands | CNS infection | Negative | 58 |
| Singapore | Mixed- 2 cases | Negative | 5 |
| Singapore | Gastrointestinal | Positive | 59 |
| Singapore | Laryngeal | Negative | 60 |
| Singapore | Disseminated | Negative | 61 |
| Singapore | Adrenal- 2 cases | Negative | 62 |
| Singapore | Disseminated- 2 cases | Positive | 63 |
| Singapore | Disseminated | Negative | 64 |
| Singapore | Oral | Negative | 65 |
| Singapore | Pulmonary | Negative | 66 |
| Singapore | Disseminated | Negative | 67 |
| Singapore | Pulmonary | Negative | 68 |
| Singapore | Disseminated | Negative | 69 |
| Singapore | Disseminated- 6 cases | Positive | 70 |
| Philippines | Disseminated | Negative | 71 |
| Philippines | Disseminated | Negative | 72 |
| Philippines | Disseminated | Unknown | 73 |
| Philippines | Disseminated | Unknown | 74 |
| Philippines | Disseminated- 9 cases | Unknown | 75 |
| Philippines | Pulmonary | Negative | 76 |
| Thailand | Mixed- 2 cases | Negative | 5 |
| Thailand | Mixed- 57 cases | Mixed | 77 |
| Thailand | Disseminated- 32 cases | Positive | 78 |
| Thailand | Endocarditis | Negative | 79 |
| Thailand | Disseminated | Negative | 80 |
| Thailand | Disseminated- 36 cases | Positive | 81 |
| Thailand | Disseminated | Negative | 82 |
| Thailand | Adrenal- 7 cases | Negative | 83 |
| Thailand | Disseminated- 5 cases | Negative | 84 |
| Thailand | Mixed- 32 cases | Mixed | 85 |
| Thailand | Disseminated- 3 cases | Positive | 86 |
| Thailand | Disseminated- 2 cases | Negative | 87 |
| Thailand | Unknown | Positive | 88 |
| Thailand | Hepatic- 6 cases | Positive | 89 |
| Thailand | Gastrointestinal | Positive | 90 |
| Thailand | Oral- 5 cases | Positive | 91 |
| Thailand | Oral- 2 cases | Positive | 92 |
| Thailand | Oral- 2 cases | Positive | 93 |
| Thailand | Pulmonary | Negative | 94 |
| Thailand | Disseminated | Positive | 95 |
| Thailand | Mucocutaneous- 2 cases | Negative | 96 |
| Potentially Thailand | Disseminated | Negative | 97 |
| Thailand | Disseminated- 2 cases | Positive | 98 |
| Thailand | Laryngeal | Negative | 99 |
| Thailand | Disseminated | Negative | 100 |
| Thailand | Disseminated | Negative | 101 |
| Thailand | Disseminated | Positive | 102 |
| Thailand | Oral | Positive | 103 |
| Thailand | Hepatic | Negative | 104 |
| Thailand | Mixed- 4 cases | Negative | 105 |
| Thailand | Disseminated | Negative | 106 |
| Thailand | Disseminated | Positive | 107 |
| Thailand | Pulmonary | Negative | 108 |
| Thailand | Myositis | Positive | 109 |
| Thailand | Colitis | Positive | 110 |
| Thailand | Mixed- 2 cases | Positive | 111 |
| Thailand | Mixed- 13 cases | Unknown | 112 |
| Thailand | Disseminated | Positive | 4 |
| Vietnam | Oral | Negative | 113 |
| Vietnam | Disseminated | Negative | 114 |
| Vietnam | Disseminated | Negative | 115 |
| Potentially Vietnam | Pulmonary | Negative | 116 |
| Vietnam | Mixed- 2 cases | Negative | 5 |

‘Potentially’ denotes a case in which histoplasmosis was potentially contracted in the noted South East Asian country but it is equally or more likely the infection occurred in an endemic country outside of South East Asia.

References

1. Pilsczek FH. Case histories of infectious disease management in developing countries: Phnom Penh and Kabul. Revista da Sociedade Brasileira de Medicina Tropical. 2009 Oct;42(5):477-83.
2. Pineau S, Talarmin JP, Morio F, Grossi O, Boutoille D, Leaute F, Le PP, Gay-Andrieu F, Miegeville M, Raffi F. Contribution of molecular biology and Aspergillus galactomannan antigen assay for the diagnosis of histoplasmosis. Medecine et maladies infectieuses. 2010 Sep;40(9):541-3.
3. Navy KB, Prey SP, Lynen L, Sovanna P, Bell JD, Harwell JI. Case report. The first reported cases of disseminated histoplasmosis in Cambodia, complicated by multiple opportunistic infections. Southeast Asian journal of tropical medicine and public health. 2005;36(5):1272.
4. Bourgeois N, Douard-Enault C, Reynes J, Lechiche C, Basset D, Rispail P, Lachaud L. Seven imported histoplasmosis cases due to Histoplasma capsulatum var. capsulatum: from few weeks to more than three decades asymptomatic period. Journal de Mycologie Médicale/Journal of Medical Mycology. 2011 Mar 1;21(1):19-23.
5. Randhawa HS. Occurrence of histoplasmosis in Asia. Mycopathologia et mycologia applicata. 1970 Sep 1;41(1-2):75-89.
6. Pratiwi W. Hubungan Pola Makan dengan Gastritis pada Remaja di Pondok Pesantren Daar El-Qolam Gintung, Jayanti, Tangerang. 2013.
7. Anggorowati N, Sulistyaningsih RC, Ghozali A, Subronto YW. Disseminated Histoplasmosis in an Indonesian HIV-Positive Patient: A Case Diagnosed by Fine Needle Aspiration Cytology. Acta Medica Indonesiana. 2018 Jan 19;49(4):360.
8. Sirait SP, Bramono K, Hermanto N. Correlation of CD 4 counts with clinical and histopathological findings in disseminated histoplasmosis: a 10‐year retrospective study. International journal of dermatology. 2017 Sep;56(9):926-31.
9. Azhar JK, Jacqueline HS, Tony LK, Tan BH, Steven JM. Bilateral adrenal histoplasmosis: endoscopic ultrasound-guided fine needle aspiration as a method of diagnosis and assessment. The Medical journal of Malaysia. 2011 Dec;66(5):504-6.
10. Wee EW, Lim SG, Wee A, Chai LY. Disseminated histoplasmosis presenting as fever and jaundice. Annals of the Academy of Medicine, Singapore. 2009 Aug;38(8):739.
11. Dwiyana RF, Rowawi R, Lestari M, Alisjahbana B, van der Ven AJ, Djajakusumah TS. Skin disorders in HIV-infected patients from West Java. Acta Med Indones. 2009 Jul;41(Suppl 1):18-22.
12. Smeets LC, Lestrade PJ, Schneeberger PM. Hoarseness in a recent visitor to the tropics through infection of the larynx by Histoplasma capsulatum. Nederlands tijdschrift voor geneeskunde. 2005 Mar;149(12):657-9.
13. Grosse G, Heise W, Staib F. Histoplasmosis of the skin as an initial opportunistic infection in AIDS. Deutsche medizinische Wochenschrift (1946). 1993 Oct;118(43):1555-60.
14. Joe LK, Tjokronegoro S, ENG NT, Fok LG. A case of progressive histoplasmosis in Indonesia. Locum. Med. geogr. trop.. 1956;5(2).
15. Wang TL, Cheah JS, Holmberg K. Case report and review of disseminated histoplasmosis in South‐East Asia: clinical and epidemiological implications. Tropical Medicine & International Health. 1996 Feb;1(1):35-42.
16. Lindeboom GA, HOOGENDIJX J, HOOGENDIJKVAN D. Histoplasmosis in Java. Documenta de medicina geographica et tropica. 1956;8(4):327-4.
17. Mead GE, Wilks D, McLaren K, Fergusson RJ. Oral histoplasmosis: a case report. Journal of Infection. 1998 Jul 1;37(1):73-5.
18. Dowe JB, Graham CS, BROWN S, DURIE EB. A case of histoplasmosis. Medical Journal of Australia. 1953(5).
19. Hausman R, Hiemstra S. A Case of Tuberculosis and Histoplasmosis. Medisch Maandblad. 1949;2(12):369-72.
20. KIRSCH E. Observations on a Case of Histoplasmosis. Zeitschrift fur Tropenmedizin und Parasitologie. 1951;3(1):86-93.
21. Swart H, HOEFNAGELS K, Pijpers PM, Stolte BH, Stolte JB. A case of histoplasmosis. Nederlands Tidjschrift voor Geneeskunde. 1971;115(4):155-7.
22. Müller H. Histoplasmosis in East Java. Geneeskundig Tijdschrift voor Nederlandsche-Indie. 1932;72(14).
23. Wahyuningsih R, Touch biopsy: A diagnosis method for Systemic Mycoses of AIDS Patients in Limited Facilities Laboratory. Poster, ISHAM 2015, Melbourne Australia
24. Bakker PM, Kuilman J. Isolated histoplasmosis of the larynx. Tijdschrift voor Diergeneeskunde. 1970;114(37):1530-33.
25. Upadana N, Suryawati N, Saputra H. PRIMARY CUTANEOUS HISTOPLASMOSIS PADA PASIEN DENGAN INFEKSI HUMAN IMMUNODEFICIENCY VIRUS (HIV).
26. Lestari S, Niode NJ, Nugroho A, Bernardus J, Meilany FD, Parasitologi D, Anatomi DP. HISTOPLASMOSIS DISEMINATA AKUT PADA PASIEN AIDS.
27. Jordan AS, Chavada R, Nagendra V, McNeil HP, Kociuba K, Gibson KA. A budding surprise from the joint. The Medical Journal of Australia. 2013 Nov 18;199(10):700-1.
28. Hu AS, Hu AS, Hu CH. Histoplasmosis with addisonian crisis: Call for bird control. Med J Malaysia. 2015 Apr 1;70(2).
29. Wahab NA, Mohd R, Zainudin S, Kamaruddin NA. Adrenal involvement in histoplasmosis. EXCLI journal. 2013;12:1.
30. Koh PS, Roslani AC, Vimal KV, Shariman M, Umasangar R, Lewellyn R. Concurrent amoebic and histoplasma colitis: A rare cause of massive lower gastrointestinal bleeding. World Journal of Gastroenterology: WJG. 2010 Mar 14;16(10):1296.
31. Ohno H, Ogata Y, Suguro H, Yokota S, Watanabe A, Kamei K, Yamagoe S, Ishida-Okawara A, Kaneko Y, Horino A, Yamane K. An outbreak of histoplasmosis among healthy young Japanese women after traveling to Southeast Asia. Internal Medicine. 2010;49(5):491-5.
32. Hasmoni MH, Shah M, Shah A, Ayoub S, Lau SH, Rashid A, Amran M. Disseminated histoplasmosis presenting with chronic ulcerative tongue lesions in a patient with diabetes. BMJ case reports. 2010.
33. Lian A, Lim Y, Heng L, Sim B, Nissapatorn V, Lee C. AIDS-defining illnesses: a comparison between before and after commencement of highly active antiretroviral therapy (HAART). Current HIV research. 2007 Sep 1;5(5):484-9.
34. Subramaniam S, Abdullah AH, Hairuzah I. Histoplasmosis of the Larynx. Medical Journal of Malaysia. 2005 Aug;60(3):386.
35. Nissapatorn V, Lee CK, Rohela M, Anuar AK. Spectrum of opportunistic infections among HIV-infected patients in Malaysia. Malay. 2003;70:34-2.
36. Rahman MT, Bakar NH, Philip R, Shamsudin AR. Oral histoplasmosis presenting as oral ulcer in a non-HIV patient. Southeast Asian J Trop Med Public Health. 2004 Jun;35(2):388-90.
37. Lachmanan SR, Haniza O, Hisham AN, Subramaniam J, Merican I. Histoplasmosis presenting with progressively worsening backache--a case report. Annals of the Academy of Medicine, Singapore. 2001 Nov;30(6):656-8.
38. Hasliza M, Nur Atiqah NA, Lim CB, Hussain IH. Disseminated histoplasmosis in a non-immunocompromised child. Med J Malaysia. 1999 Mar;54(1):120-4.
39. Tan HJ, Cheong I, Muhaizan WM. Disseminated histoplasmosis in AIDS: a report of three patients. The Medical journal of Malaysia. 2000 Jun;55(2):259-62.
40. Jing W, Ismail R. Mucocutaneous manifestations of HIV infection: a retrospective analysis of 145 cases in a Chinese population in Malaysia. International journal of dermatology. 1999 Jun;38(6):457-63.
41. Ng KH, Siar CH. Review of oral histoplasmosis in Malaysians. Oral Surgery, Oral Medicine, Oral Pathology, Oral Radiology and Endodontics. 1996 Mar 1;81(3):303-7.
42. Zainudin BM, Kassim F, Annuar NM, Lim CS, Ghazali AK, Murad Z. Disseminated histoplasmosis presenting with ileal perforation in a renal transplant recipient. The Journal of tropical medicine and hygiene. 1992 Aug;95(4):276-9.
43. Liam CK, Chua CT, Pathmanathan R. Disseminated histoplasmosis presenting as a non-healing tongue ulcer. Singap Med J. 1990 Jun;31:286-8.
44. Meah F. Adrenal histoplasmosis in Cushing's syndrome with bilateral adrenocortical nodular hyperplasia.
45. Eravelly J, Ramanathan K, Eapen JS. Histoplasmosis: long term remission following treatment with low dose amphotericin-B. Medical Journal of Malaysia. 1975;30(1):59-62.
46. Jayalakshmi P, Goh KL, Soo‐Hoo TS, Daud A. Disseminated histoplasmosis presenting as penile ulcer. Australian and New Zealand journal of medicine. 1990 Apr;20(2):175-6.
47. Soo‐Hoo TS, Adam BA, Yusof D. Disseminated primary cutaneous histoplasmosis. Australasian Journal of Dermatology. 1980 Aug;21(2):105-7.
48. Chan KS, Looi LM, Chan SP. Disseminated histoplasmosis mimicking miliary tuberculosis: a case report. The Malaysian journal of pathology. 1993 Dec;15(2):155-8.
49. Fujio J, Nishimura K, Miyaji M. Epidemiological survey of the imported mycoses in Japan. Nippon Ishinkin Gakkai Zasshi. 1999 Apr 30;40(2):103-9.
50. Vijayananthan A, Abdullah BJ, Chan SP. Fine needle aspiration biopsy in the diagnosis of disseminated histoplasmosis of the adrenal glands. JOURNAL-HONG KONG COLLEGE OF RADIOLOGISTS. 2003;6:152-4.
51. Shahar MA, Wahab NA, AbAziz A, Marzuki OA, Rusly NM, Azmi KN. Adrenal insufficiency resulting from Histoplasmosis infiltration in an immunocompetent patient. Brunei International Medical Journal. 2015;11(5):261-4.
52. Mlawa G, Deore M, Nageshwaran S. A rare case of adrenal histoplasmosis presenting as adrenal insufficiency and hypogonadism following a visit to bat caves. In17th European Congress of Endocrinology 2015 May 1 (Vol. 37). BioScientifica.
53. Madden M, Kennedy JD, Hitchcock HT, O'Beirn D. Chronic pulmonary histoplasmosis in an irishman. Thorax. 1981 Sep;36(9):705.
54. Ponnampalam JT, Kumar G. Histoplasmosis in Malaya, oro-pharyngeal and disseminated: Treatment with amphotericin B. Transactions of the Royal Society of Tropical Medicine and Hygiene. 1966 Jan 1;60(1):92-6.
55. Sharma R, Lipi L, Gajendra S, Mohapatra I, Goel RK, Duggal R, Mishra SR, Gautam D. Gastrointestinal Histoplasmosis: A Case Series. International journal of surgical pathology. 2017 Oct;25(7):592-8.
56. Murty OP. Cystic tumor of papillary muscle of heart: a rare finding in sudden death. The American journal of forensic medicine and pathology. 2009 Jun 1;30(2):201-3.
57. Hung MN, Sun HY, Hsueh PR, Hung CC, Chang SC. Meningitis due to Histoplasma capsulatum and Mycobacterium tuberculosis in a returned traveler with acquired immunodeficiency syndrome. Journal of the Formosan Medical Association= Taiwan yi zhi. 2005 Nov;104(11):860-3.
58. Wheat J, Myint T, Guo Y, Kemmer P, Hage C, Terry C, Azar MM, Riddell J, Ender P, Chen S, Shehab K. Central nervous system histoplasmosis: Multicenter retrospective study on clinical features, diagnostic approach and outcome of treatment. Medicine. 2018 Mar;97(13).
59. Mukherjee P, Chawla A, Lang TU. A Rare Cause of a Positive Fecal Occult Blood Test. Gastroenterology. 2016 Aug 1;151(2):248-9.
60. Teoh JW, Hassan F, Mohamad Yunus MR. Laryngeal histoplasmosis: an occupational hazard. Singapore Med J. 2013 Oct 1;54(10):e208-10.
61. Yap ES, Chee YL. Disseminated H istoplasma capsulatum diagnosed on peripheral blood film. European journal of haematology. 2013 May;90(5):440-.
62. Mukherjee JJ, Villa ML, Tan L, Lee KO. Bilateral adrenal masses due to histoplasmosis. The Journal of Clinical Endocrinology & Metabolism. 2005 Dec 1;90(12):6725-6.
63. Chee L. Histoplasmosis in the head and neck. Annals of the Academy of Medicine, Singapore. 1997 Jul;26(4):510-3.
64. Wong SY, Allen DM. Transmission of disseminated histoplasmosis via cadaveric renal transplantation: case report. Clinical infectious diseases. 1992 Jan 1;14(1):232-4.
65. Loh FC, Yeo JF, Tan WC, Kumarasinghe G. Histoplasmosis presenting as hyperplastic gingival lesion. Journal of Oral Pathology & Medicine. 1989 Oct;18(9):533-6.
66. Kunaratnam N, Tan KK, Adams M. A case of histoplasmosis in Singapore. Singapore Med J. 1960 Sep;1:99-105.
67. Tong P, Tan WC, Pang M. Sporadic disseminated histoplasmosis simulating miliary tuberculosis. British medical journal (Clinical research ed.). 1983 Sep 17;287(6395):822.
68. SUPRAMANIAM J. The first reported case of broncholithiasis in Singapore. Singapore Med J. 1962 Jun;3:94-7.
69. Ng Y, Quah TC. Acute disseminated histoplasmosis in an immunocompetent child: A case report and review of the literature.
70. Bellamy R, Sangeetha S, Paton NI. AIDS-defining illnesses among patients with HIV in Singapore, 1985 to 2001: results from the Singapore HIV Observational Cohort Study (SHOCS). BMC infectious diseases. 2004 Dec;4(1):47.
71. Azar MM, Malinis MF. Disseminated histoplasmosis with skin lesions and osteomyelitis in a patient from the Philippines. The American journal of tropical medicine and hygiene. 2016 Jul 6;95(1):70-4.
72. Navarro EE, Tupasi TE, Verallo VM, Romero RC, Tuazon CU. Disseminated histoplasmosis with unusual cutaneous lesions in a patient from the Philippines. The American journal of tropical medicine and hygiene. 1992 Feb 1;46(2):141-5.
73. Palencia RJ, Aniceto EG, Pena AC. Fever of unknown origin with hepatomegaly due to Histoplasma capsulatum: case report. Phil J Microbiol Infect Dis. 1990 Jul;19:63-7.
74. Navarra ST, Mateo MO, Bulmer CS, Torralba LD, Javate R. A culturally proven case of disseminated histoplasmosis in the Philippines. Phil. J. Int. Med. 1992 Mar;30:69-77.
75. De Luna-Tacastacas J, Bacorro EA, Berba RP. A 47 Year Old Male with Multiple Ulcerated Skin Plaques: A Case Report on Disseminated Histoplasmosis and A Review of Eight Previously Reported Cases of Histoplasmosis in the Philippines.
76. Gulay CB, Albay Jr A. Interstitial Lung Disease Due To Histoplasmosis: A Case Report. InC44. DIFFUSE PARENCHYMAL LUNG DISEASE: CASE REPORTS 2016 May (pp. A5132-A5132). American Thoracic Society. Buraphat P, Pongpirul W, Suttha P. Disseminated histoplasmosis in a patient with HIV diagnosed by simple bedside investigations. BMJ Case Rep. 2017 Jun 24;2017.
77. Wongprommek P, Chayakulkeeree M. Clinical Characteristics of Histoplasmosis in Siriraj Hospital. Journal of the Medical Association of Thailand= Chotmaihet thangphaet. 2016 Mar;99(3):257-61.
78. Norkaew T, Ohno H, Sriburee P, Tanabe K, Tharavichitkul P, Takarn P, Puengchan T, Bumrungsri S, Miyazaki Y. Detection of environmental sources of Histoplasma capsulatum in Chiang Mai, Thailand, by nested PCR. Mycopathologia. 2013 Dec 1;176(5-6):395-402.
79. Lorchirachonkul N, Foongladda S, Ruangchira-Urai R, Chayakulkeeree M. Prosthetic valve endocarditis caused by Histoplasma capsulatum: the first case report in Thailand. Journal of the Medical Association of Thailand= Chotmaihet thangphaet. 2013 Feb;96:S262-5.
80. Kathuria S, Capoor MR, Yadav S, Singh A, Ramesh V. Disseminated histoplasmosis in an apparently immunocompetent individual from north India: a case report and review. Medical mycology. 2013 Oct 1;51(7):774-8.
81. Rangwala F, Putcharoen O, Bowonwatanuwong C, Edwards-Jackson N, Kramomthong S, Kim JH, Corey GR, Ananworanich J. Histoplasmosis and penicilliosis among HIV-infected Thai patients: a retrospective review. Southeast Asian J Trop Med Public Health. 2012 Mar 1;43(2):436-41.
82. Wisanuyotin S, Jiravuttipong A, Siritunyaporn S. Disseminated histoplasmosis in a renal transplant child. Southeast Asian J Trop Med Public Health. 2012 Jul 1;43:992-6.
83. Larbcharoensub N, Boonsakan P, Aroonroch R, Rochanawutanon M, Nitiyanant P, Phongkitkarun S, Poonvutikul S, Watcharananan SP, Ngarmukos C. Adrenal histoplasmosis: a case series and review of the literature. Southeast Asian Journal of Tropical Medicineand Public Health. 2011 Jul 1;42(4):920.
84. Chetchotisakd P, Kiertiburanakul S, Mootsikapun P, Assanasen S, Chaiwarith R, Anunnatsiri S. Disseminated nontuberculous mycobacterial infection in patients who are not infected with HIV in Thailand. Clinical infectious diseases. 2007 Aug 15;45(4):421-7.
85. Mootsikapun P, Srikulbutr S. Histoplasmosis and penicilliosis: comparison of clinical features, laboratory findings and outcome. International journal of infectious diseases. 2006 Jan 1;10(1):66-71.
86. Anekthananon T, Ratanasuwan W, Techasathit W, Rongrungruang Y, Suwanagool S. HIV infection/acquired immunodeficiency syndrome at Siriraj Hospital, 2002: time for secondary prevention. Journal of the Medical Association of Thailand= Chotmaihet thangphaet. 2004 Feb;87(2):173-9.
87. Roubsanthisuk W, Sriussadaporn S, Phoojaroenchanachai M, Peerapatdit T, Nitiyanant W, Vannasaeng S, Vichayanrat A, Vawesorn N, Parichatikanond P, Homsanit M. Primary adrenal insufficiency caused by disseminated histoplasmosis: report of two cases. Endocrine Practice. 2002 May 1;8(3):237-41.
88. Tamura M, Kasuga T, Watanabe K, Katsu M, Mikami Y, Nishimura K. Phylogenetic characterization of Histoplasma capsulatum strains based on ITS region sequences, including two new strains from Thai and Chinese patients in Japan. Nippon Ishinkin Gakkai Zasshi. 2002 Jan 30;43(1):11-9.
89. Piratvisuth T, Siripaitoon P, Sriplug H, Ovartlarnporn B. Findings and benefit of liver biopsies in 46 patients infected with human immunodeficiency virus. Journal of gastroenterology and hepatology. 1999 Feb;14(2):146-9.
90. Hung CC, Wong JM, Hsueh PR, Hsieh SM, Chen MY. Intestinal obstruction and peritonitis resulting from gastrointestinal histoplasmosis in an AIDS patient. Journal of the Formosan Medical Association= Taiwan yi zhi. 1998 Aug;97(8):577-80.
91. Nittayananta W, Chungpanich S. Oral lesions in a group of Thai people with AIDS. Oral diseases. 1997 May;3(S1):S41-5.
92. Nittayananta W, Kumplanont P, Srisintorn S, Akkayanont P, Chungpanich S, Teanpaisan R, Chareonwatanan M, Nuntanaranont T. Oral histoplasmosis associated with candidiasis in HIV-infected patients: a report of two cases. British dental journal. 1997 Apr 26;182(8):309.
93. Nittayananta W, Jealae S, Chungpanich S. Oral lesions in Thai heterosexual AIDS patients: a preliminary study. British dental journal. 1997 Mar 22;182(6):219.
94. Hirsch D, Leupold W, Rupprecht E. Pulmonary histoplasmoma after travel abroad. Pneumologie (Stuttgart, Germany). 1996 Mar;50(3):242-4.
95. Poinsilpa KP, Tantawichien T. Disseminated histoplasmosis in a young Thai male. Journal of travel medicine. 1994 Mar;1(1):57-.
96. Bhamarapravati N, Balankura P, Sekhonrit C. Histoplasmosis in Thailand: report of two cases diagnosed by biopsy and culture. The American journal of tropical medicine and hygiene. 1963 May 1;12(3):393-7.
97. Schulze AB, Heptner B, Kessler T, Baumgarten B, Stoica V, Mohr M, Wiewrodt R, Warneke VS, Hartmann W, Wüllenweber J, Schülke C. Progressive histoplasmosis with hemophagocytic lymphohistiocytosis and epithelioid cell granulomatosis: a case report and review of the literature. European journal of haematology. 2017 Jul;99(1):91-100.
98. Siwamogstham P, Reichart PA, Thosaporn W, Prapamonton N. Oral histoplasmosis associated with HIV Disease: Report of two cases from Thailand. Oral Biosci Med. 2005;4:253-7.
99. Imvidhya SA, Vichavasiri AP, Supiyapan PA, Chantarakul NI. Histoplasmosis of the larynx: report of a case. Journal of the Medical Association of Thailand= Chotmaihet thangphaet. 1985 Sep;68(9):485.
100. Pongsapich W, Samphaiboon C, Sukpanichnant S. PP034: Oral ulcer resembles SCCA as a presenting symptom of systemic histoplasmosis in an immunocompetent patient: A case report. Oral Oncology. 2013 May 1;49:S105.
101. Rungruxsirivorn S. Disseminated histoplasmosis: a case report. Journal of the Medical Association of Thailand. 1980;63(4):215-9.
102. Summachiwakij S, Tungsubutra W, Koomanachai P, Charoenratanakul S. Chylous ascites and chylothorax due to constrictive pericarditis in a patient infected with HIV: a case report. Journal of medical case reports. 2012 Dec;6(1):163.
103. Nittayananta W, Kumplanont P, Chungpanich S, Mitarnun W. Oral histoplasmosis as the first evidence of AIDS. JOURNAL-DENTAL ASSOCIATION OF THAILAND. 1996;46:58-62.
104. Phoompoung P, Chayakulkeeree M, Ngamskulrungroj P, Pongpaibul A. Asymptomatic Histoplasma Pylephlebitis in an Orthotopic Liver Transplant Recipient: A Case Report and Literature Review. Mycopathologia. 2018 May 22:1-4.
105. Sanpitak P, Srisuwan S, Panas K, Limpatakmongkol P. Histoplasmosis: Four cases from Northern Provines of Thailand. Chiang Mai Medical Journal-เชียงใหม่ เวช สาร. 1973;12(4):305-12.
106. Panthuwong S, Siripaitoon P, Silpapojakul K. Fatal disseminated histoplasmosis in a previously healthy person.
107. Supparatpinyo K, Kwangsuksatith C, Hirunsri P, Uthammachai C, Sirisanthana T. Systemic Mycosis Caused By Cryptococcus neoformans, Penicillium marneffei, and Histoplasma capsulatum.
108. Chantarakul N, Parichatikanond P. Histoplasmoma of the lung. A necropsy: case report. Journal of the Medical Association of Thailand. 1977;60(8):379-82.
109. Nimitvilai S, Thammaprasert W, Vinyuvat S. HISTOPLASMOSIS MYOSITIS: A CASE REPORT AND.
110. Manatsathit S, Tansupasawasdikul S, Wanachiwanawin D, Setawarin S, Suwanagool P, Prakasvejakit S, Leelakusolwong S, Eampokalap B, Kachintorn U. Causes of chronic diarrhea in patients with AIDS in Thailand: a prospective clinical and microbiological study. Journal of gastroenterology. 1996 Aug 1;31(4):533-7.
111. Inverarity D, Bradshaw Q, Wright P, Grant A. The spectrum of HIV-related disease in rural Central Thailand. Southeast Asian journal of tropical medicine and public health. 2002 Dec;33(4):822-31.
112. Chantharit P, Watcharananan S, Sungkanuparph S. Comparison of clinical characteristics and survival among patients with cryptococcosis, histoplasmosis, and penicilliosis in Ramathibodi Hospital. International Journal of Antimicrobial Agents. 2013 Jun 1;42:S120.
113. Smith R, Lee A, Gottlieb T, Eckstein R, Meng L. Another case of histoplasmosis in a non‐endemic area. Pathology. 2006 Jan 1;38(4):378-80.
114. MAYDAT L. The first case of generalized histoplasmosis in South Vietnam. Bulletin de la Societe de pathologie exotique et de ses filiales. 1962;55:35.
115. Radin DR. Disseminated histoplasmosis: abdominal CT findings in 16 patients. AJR. American journal of roentgenology. 1991 Nov;157(5):955-8.
116. Shub C, Alexander BB. Suspected subclinical mycotic disease: a case report. Military medicine. 1971 Jul 1;136(7):622-3.
