# Supplementary material for: Mapping histoplasmosis in South East Asia – implications for diagnosis in AIDS
Source: Emerg Microbes Infect. 2019 Jul 31;8(1):1139–45. doi: 10.1080/22221751.2019.1644539 (PMC6711083; doi:10.1080/22221751.2019.1644539)
Supplement: Supplemental Material [file TEMI_A_1644539_SM4495.zip › Baker_Figure_S1_Revised.docx]

Figure S1. Literature searching process.

HDAS search of PubMed and Medline databases (duplicates removed)

123 publications including 320 cases

Google scholar results:

First 500 results reviewed

With duplicates removed- 53 cases

373 individual cases of histoplasmosis

Search of Cochrane Library- No articles.

Additional cases from scanning selected bibliographies in the Indonesian language

Data from governmental public health authorities

407 individual cases of histoplasmosis

Data from Thai public health discarded due to lack of detail

Flow chart showing literature search process.
